# *N*-Chlorotaurine Solutions as Agents for Infusion Detoxification Therapy: Preclinical Studies

**DOI:** 10.3390/ijms25158345

**Published:** 2024-07-30

**Authors:** Bohdan Murashevych, Gennadii Bilenkyi, Dmitry Girenko, Emil Bilenkyi

**Affiliations:** 1 Department of Biochemistry and Medical Chemistry, Dnipro State Medical University, 49044 Dnipro, Ukraine; 2 Clinical Hospital of Emergency Medical Care of the Dnipro City Council, 65 Volodymyra Antonovycha Str., 49000 Dnipro, Ukraine; 3 Department of Physical Chemistry, Ukrainian State University of Chemical Technology, 8 Gagarina Ave., 49005 Dnipro, Ukraine; dvgir1970@gmail.com

**Keywords:** *N*-chlorotaurine, active chlorine, *N*-chloramines, antimicrobial, infusion, detoxification therapy

## Abstract

*N*-chlorotaurine (NCT) is a broad-spectrum antimicrobial agent with outstanding tolerability, effective for topical and inhalation use. This paper presents the results of studies of single and repeated intravenous infusions of NCT to laboratory animals. The studies were conducted on female Wistar Han rats. The effect of NCT infusions on the general condition, behavioral reactions, main biochemical and hematological parameters, hemocoagulation system, cardiovascular system, and on the condition of the internal organs was studied. It was found that NCT infusions do not reveal deviations in the studied parameters that could indicate a toxic effect. The estimated LD50 is more than 80 mg/kg. In a subchronic experiment, a statistically significant decrease in cholesterol (by up to 11%), glucose (by up to 15%) and excess bases (up to four times) in the blood, and an increase in heart rate (by up to 31%) and frequency of defecations (by up to 35%), as well as pronounced antiplatelet effect, were found. In animals with simulated endotoxicosis, a decrease in the cytolysis and oxidative stress markers was observed. Such effects are caused by both chlorine-active compounds and taurine.The results obtained indicate broad prospects for the use of NCT solutions as an infusion detoxifying agent.

## 1. Introduction

Active chlorine compounds (hypochlorous acid, its salts, as well as *N*-chloramines of various structures, including those immobilized on polymer carriers) have long been known as powerful microbiocidal agents and are widely used for sanitary and hygienic measures and in water treatment [1,2,3,4,5]. The COVID-19 pandemic also stimulated numerous studies in the direction of the use of such substances for the prevention and therapy of infectious diseases: from the treatment of infected wounds to inhalations for acute respiratory distress syndromes of various etiologies [6,7,8]. The undeniable advantage of chlorine-active compounds is their wide range of microbiocidal activity: they are effective against bacteria, fungi, viruses, and prions, while their mechanism of action, which is based on oxidizing and chlorinating properties, is systemic and does not involve the possibility of resistance [9,10,11,12,13]. In addition, basic industrial preparations (sodium hypochlorite, chloramines B and T, etc.) are quite simple to synthesize and cheap. At the same time, their medical use is limited by insufficient stability and the presence of certain toxic and irritating impurities (sodium chlorate, sodium hydroxide, etc.).

*N*-chlorotaurine (NCT), solutions of which have been widely studied in the works of Nagl, Gottardi and colleagues, is a separate representative of chlorine-active compounds that is especially promising for use in medicine [14]. Although this substance has a slightly lower redox potential than classic sodium hypochlorite and hypochlorous acid, it remains an effective antimicrobial agent, while its toxicity is lower [15,16,17]. It can be obtained both in the form of a solution through the interaction of free taurine with chlorinating agents (mostly sodium hypochlorite) [18] and in crystalline form using a specially developed technology [19]. In addition, in situ synthesis is also possible using polymer materials with immobilized *N*-chlorosulfonamide groups as a source of active chlorine [20,21]. Thus, it is possible to obtain highly pure dosage forms for various ways of administrating them into the body. However, the prospects for the external use of NCT solutions as an antimicrobial agent were mainly investigated [22]. Their effectiveness in the treatment of various skin diseases, such as non-healing wounds [23], acne vulgaris [24], herpes zoster [25], etc., has been proven. In addition, recent studies demonstrate the beneficial effect of NCT inhalations in the early stages of COVID-19 and other viral respiratory infections, having high tolerability [26]. It has also been shown that NCT can inhibit the synthesis of IL-6 and IL-8, which theoretically predicts the feasibility of its use in the treatment of various hyper-inflammatory diseases [27,28].

At the same time, broad prospects for the use of chlorine-active compounds for intravenous administration are obvious. In Ukraine and CIS countries, the use of hypochlorous acid and its salts as infusion therapeutics is called “indirect electrochemical detoxification” [29,30,31]. Its use makes it possible to fight not only with pathogens of a microbiological nature but also to neutralize a large number of endogenous toxins inherent in various pathological conditions. This is achieved due to their oxidation and the related hydrophilicization, which facilitates their excretion by natural means. Thus, a complex therapeutic effect can be ensured. It has been shown that administration of 0.03–0.06% sodium hypochlorite solution improves biochemical and hematological parameters in patients with urological diseases [32,33], acute cholangitis [34], multiple melanoma [35], in patients after multi-organ operations [36,37], etc. Intravenous administration of more active hypochlorous acid (“neutral electrolyzed saline”) instead of alkaline sodium hypochlorite is also being actively investigated, for example, for the treatment of rheumatoid arthritis [38], as well as in the treatment of COVID-19 [39]. In addition, the administration of such solutions can be considered as a detoxifier in case of poisoning with ethanol and other psychotropic substances [40,41]. It is obvious that the similarity of the mechanism of action of hypochlorites and NCT allows predicting similar effects when the latter is administered intravenously. At the same time, its use might have a number of advantages: lower reactivity and, accordingly, a longer lifetime in biological fluids allows NCT to spread through the vessels over a greater distance, the local toxic effect and circulatory disorders should be smaller, and in addition, taurine itself as a product of NCT metabolism will provide additional therapeutic effects (anti-inflammatory, energetic, antioxidant, etc.). Thus, NCT solutions can be effective and affordable multipurpose infusion drugs. This article describes the results of preclinical studies of NCT solutions during their single and repeated intravenous administration to intact animals and animals with simulated endotoxicosis.

## 2. Results

When the NCT solution of the specified composition was administered to laboratory animals, no significant anxiety in the rats was noted, which could be caused by sharp pain sensations. At the same time, the administration of the reference sodium hypochlorite solution was objectively not comfortable for the animal. In all experiments, no visible damage to the vessels and tissues of the rat tails was observed, even in cases where a large volume of the drug required multiple daily injections. These facts indirectly indicate the absence of a traumatic effect of NCT on blood vessels.

### 2.1. Results of the Acute Toxicity Study of Intravenous Administration of NCT Solution

The death of animals from the experimental, reference, and control groups was not observed during the entire period of the experiment. It was noted that 30 min after the administration of NCT solution in the volume of 1.0 CBV, one of the six animals of the corresponding group showed slight retardation and hypodynamia. However, within 90 min, the rat’s condition returned to normal. In the group of rats that received NCT in a volume of 2.0 CBV, intensive grooming and increased salivation against the background of general hypodynamia were observed in two rats; however, 4 h after the last administration of the drug, these symptoms disappeared. At the same time, similar changes in the general condition were observed in three out of six animals that were injected with a solution of 0.06% NaOCl in the amount of 1.0 CBV and in all animals that were injected with NaOCl solution in the amount of 2.0 CBV, but all the listed symptoms disappeared within 12 h. In all animals that were injected with solutions of taurine and sodium chloride in large volumes, hypodynamia was also noted within 2–6 h. During the next 14 days of the experiment, there were no visible differences in the general condition, appearance, and behavior of the rats of the experimental and control groups: the animals were active throughout the observation period, did not show signs of anxiety, and food and water consumption remained at the same level. There were no cases of pathological changes in the skin, coat, mucous membranes, or excretory system or physiological abnormalities during the 2-week experiment in all groups. According to the results of the body weight assessment of the experimental animals, the dynamics of the indicated indicator during the 14-day observation in all groups remained within the physiological norm and did not statistically differ from the corresponding indicators of rodents in the control group. There were no changes in the macroscopic state and relative weight of the internal organs of the animals of the experimental groups compared to the control ones.

### 2.2. Results of the Subchronic Toxicity Study of Intravenous Administration of NCT Solution

No animal from the experimental or control groups died during the entire period of the experiment. The rats were active, showed no signs of anxiety, and consumed food and water at the same level as in the control group. Pathological changes in the skin, coat, mucous membranes, and work of the excretory system, as well as physiological abnormalities, were not observed during the 28 days. The dynamics of changes in the body weight of the experimental animals remained within the physiological norm and did not differ from the control group.

The results of studying indicators of unconditioned reflex behavior of laboratory rats in the “open field” after 28-day intravenous administration of NCT solution are shown in Table 1.

The data in Table 1 indicate a slight dose-dependent decrease in the activity of animals after a course of NCT administration. In addition, a dose-dependent increase in the frequency of animal defecation was observed (increase by 35% relative to the control when 0.5 CBV of NCT was administered).

The results of the assessment of hematological indicators of the peripheral blood of rats under the conditions of repeated administrations of the studied drug are presented in Table 2.

The data in Table 2 indicate the absence of statistically significant deviations in the content of basic blood cells and hemoglobin in rats of both experimental groups compared to the control group.

Biochemical indicators of blood serum of experimental animals, which characterize the functional state of the liver and kidneys in rats after long-term administration of NCT solution, are shown in Table 3.

As can be seen from Table 3, most of the studied indicators did not differ statistically in the animals of the experimental and control groups. There was a slight tendency towards a dose-dependent increase in the levels of AST (higher by 4% in animals injected with 0.5 CBV of NCT compared to controls) and ALP (increase by 9%, respectively). At the same time, a pronounced dose-dependent decrease in the level of cholesterol (by 11% in animals injected with 0.5 CBV of NCT compared to the control) and glucose (by 15% in the same groups) was observed.

The results of the study on NCT’s influence on the indicators of the coagulation link of the rats’ hemostasis system are presented in Table 4.

The data in Table 4 indicate the absence of a noticeable effect of repeated administration of NCT on the coagulation link of the rat hemostasis system.

The results of the study on the NCT solution’s effect on the indicators of the vascular–platelet link of the hemostasis system (platelet aggregation function) are presented in Table 5 and Table 6.

As can be seen, the long-term administration of NCT leads to a significant decrease in both the maximum degree and the speed of induced aggregation of platelets in experimental animals compared to the parameters of the control group, regardless of the applied inducer. The maximum degree of aggregation in animals injected with 0.2 and 0.5 CBV of NCT practically does not differ. At the same time, the rate of platelet aggregation is dosedependent. Thus, in animals injected with 0.5 CBV of NCT, this indicator decreases by 62% when induced by collagen compared to the control group, and in the case of administration of 0.2 CBV of NCT—by 48%. When 5 μM ADP is used as an inducer, the reduction is 51% and 33%, respectively.

The results of determining the acid–alkaline state of the rat blood after long-term intravenous administration of NCT are shown in Table 7.

As can be seen from Table 7, most of the determined indicators of experimental animals are not statistically different from those of the control group. Attention is drawn to the significant decrease in ABE and SBE, which directly depends on the amount of injected NCT: the ABE indicator in animals that received 0.2 CBV of NCT is 1.7 times lower and in animals that received 0.5 CBV—by 4.6 times; for SBE, such changes are 1.72 and 3.95 times, respectively.

The results of the analysis of rat urine indicators after repeated intravenous administration of NCT solution are presented in Table 8.

The data in Table 8 show that there were no significant deviations of the investigated urine parameters of the experimental animals from the animals in the control group.

The main indicators of the functioning of the cardiovascular system of rats treated with NCT for 4 weeks are presented in Table 9.

Among the indicators in Table 9, reliably significant deviations were observed only in heart rate and RR interval. At the same time, surprisingly, the influence of 0.2 CBV of NCT infusions on these parameters was greater than that of 0.5 CBV of NCT infusions. Thus, the heart rate of animals receiving NCT in a volume of 0.2 CBV increased by 31%, while for animals after administration of 0.5 CBV NCT the increase was 25%. The RR interval in animals of both experimental groups decreased by 15% and 14%, respectively. All indicators still remained within the physiological norm of animals of a given weight and age.

Macro- and microscopic studies did not reveal significant pathomorphological changes in the organs of the experimental animals compared to the animals of the control group. A detailed description of the research in this article is not provided due to its large volume and can be provided upon request.

### 2.3. Results of the NCT Solution Infusions to the Rats with Modeled Endotoxicosis

The results of studies of the detoxifying activity of NCT and reference drugs in conditions of simulated chronic endotoxicosis are presented in Table 10.

It follows from Table 10 that under the conditions of experimental chronic endotoxicosis caused by the administration of CCl_4_ and LPS, a significant increase in the concentrations of MDA and DC by 102% and 133%, respectively, was observed, which indicated a pronounced activation of lipoperoxidation processes. A significant—by 44%—decrease in the activity of SOD in the liver tissue was also noted, which can lead to the accumulation of a large number of toxic free radical catabolites in the blood and tissues capable of causing reactions from the immune system. A significant increase in the levels of ALT (by 3.12 times) and AST (by 2.47 times) relative to the indicators of the intact group was established, which is a marker of hepatocyte cytolysis. It was found that the repeated administration of NCT under experimental conditions had a profound effect on all the listed indicators. Thus, in comparison with the parameters of the control group, the concentrations of MDA and DC decreased significantly (by 34% and 36%, respectively), as well as the activity of transaminases (ALT by 53% and AST by 51%). The activity of SOD in the liver tissue increased by 64%. It was noted that comparable changes in the activity indicators of cytolysis, lipoperoxidation, and antioxidant protection were recorded when using sodium hypochlorite solution and much lesswhen using taurine.

## 3. Discussion

Currently, the main object of research into the pharmacological effects of NCT is its solutions obtained from the crystalline form, the synthesis of which was developed by Nagl and Gottardi [19]. Most publications describe the use of 1% NCT solution in a phosphate buffer at a pH of about 7.4 [22,23,24,25,26]. The acidity of unbuffered 1% NCT solution is about 8.2 [15]. The parameters of the NCT solution used in our study differ significantly (Table 12), which is due to a different production technology. Obtaining the NCT solution from sodium hypochlorite, which was used in this work, allows quick establishment of large-scale production, provided that the technology for obtaining the original solution of active chlorine is followed. The NaOCl solution used by us is characterized by a low content of sodium chlorate, which is known to exhibit a number of toxic effects [42], and a reduced content of free alkali (initial pH around 9.5). In addition, it is isotonic due to the presence of an appropriate amount of background electrolyte—sodium chloride, which provides the possibility of intravenous administration. Such a solution is more stable than hypochlorites obtained by the traditional “chemical” method (dissolving molecular chlorine in a concentrated alkali solution) [20], has proven itself well in veterinary medicine [43,44], and does not show a toxic effect even when administered by inhalation [45]. Thus, the composition of the NCT preparation used is similar to the solution that was administered intraperitoneally in the study of induced pneumonia in obese mice [46]. The lower concentration of the active compound is due to the need to minimize the pain effect during infusion, and the slightly alkaline pH is necessary to increase the stability of NCT and prevent the formation of *N*,*N*-dichlorotaurine and sulfoacetaldehyde during long-term storage. The formation of NCT during the synthesis of our solution was confirmed by UV spectra with a characteristic absorption peak near 250 nm [15].

Studies of the effect of a single intravenous NCT injection in rats in a wide range of concentrations, as can be seen, did not cause the death of animals even in a volume of 2.0 CBV. The observed minor deviations in the general condition of the animals are due, most likely, to large volumes of infusion solutions. However, as for the reference sodium hypochlorite, intensification of grooming and salivation can also be caused by pain. As is known, the purpose of acute toxicity tests is to establish the LD50 [47]. There are currently no established LD50 for NCT by any route of administration. In our experiment, the impossibility of significantly increasing the NCT concentration in the tested solution, due to the use of a specific starting solution of sodium hypochlorite, as well as the impossibility of administrating even larger volumes of the drug, did not allow us to establish the LD50 for it. At the same time, even for intravenous use of sodium hypochlorite, the calculated LD50 is about 33.3 mg/kg [48], which is much higher than the applied amounts of NCT (in terms of active chlorine). In addition, the lower oxidizing properties of NCT will probably lead to much lower toxicity. At this stage, we can only state that the LD50 of the NCT test solution is more than 80 mg/kg.

No significant changes in their behavior were observed when rats were injected with NCT solution periodically during the 28 days. The most obvious is an increase in the frequency of defecation, which can be explained by the effect of high concentrations of free taurine as a product of NCT metabolism [49]. With regard to hematological studies, the most important result is the absence of a decrease in the level of hemoglobin in experimental animals (Table 2), because it is known that oxidants, in particular hypochlorous acid, quickly and irreversibly lead to the destruction of heme and the accumulation of iron ions [50,51]. As for markers of hepatotoxicity (ALT and AST), their increase after a 28-day course of NCT is insignificant, especially compared to their levels against the background of simulated endotoxicosis (Table 10), which indicates the absence of hepatotoxicity of such infusions. Changes in the ALP level are somewhat more noticeable, which is consistent with the data of in vitro studies [52,53]; at the same time, our previous studies of active chlorine inhalation demonstrated, on the contrary, a decrease in the level of ALP in rats [45]. The decrease in the level of cholesterol in animals after a course of NCT is probably explained by the possibility of its oxidation by active chlorine compounds [54,55]. A wide variety of products of such oxidation/chlorination, such as oxosterols and chlorohydrins, as well as the very fact of reducing cholesterol, can lead to various positive (reduction in the risk of cardiovascular disorders [56]) and negative (arrhythmias and reduction in the contractile function of cardiomyocytes [57], inflammation [58], etc.) effects that require further study. The decrease in glucose level in the rat’s blood after a course of NCT also attracts attention. It was previously reported that chlorine-active compounds in in vitro experiments inhibit enzymes of various pathways of glucose metabolism and also contribute to insulin resistance, which in general should lead, on the contrary, to an increase in the glucose concentration in the blood of experimental animals [59,60]. The results we obtained can be explained by the dominant effect of administrated taurine (and not active chlorine), the consumption of which helps to reduce the glycemic index and fasting blood sugar [61,62].

The main conclusion that can be drawn from our studies of the rat hemostasis system after repeated administration of NCT solution (Table 4, Table 5 and Table 6) is that the drug has pronounced antiplatelet properties. Such properties of active chlorine compounds were described earlier [63,64] and can be explained by the oxidation of surface sulfhydryl groups of platelets, as well as an increase in the production of prostacyclin by the vascular wall. At the same time, some studies show opposite results [65,66]. But the effect of taurine as a metabolite of NCT is unambiguous: it significantly reduces platelet aggregation [67,68]; there are isolated studies that confirm such an effect for NCT itself [69]. Thus, the antiplatelet effect of NCT infusions is beyond doubt.

Our data on the increase in heart rate in rats after a 28-day administration of NCT solution do not contradict the works [70,71], from which we can conclude that this effect is caused by taurine. There are currently no data on the effect of chlorine-active compounds on this indicator.

Thus, repeated intravenous administration of NCT solution to rats for 28 days did not lead to critical deviations in hematological and biochemical indicators, as well as pathomorphological changes in internal organs, which would clearly indicate a pronounced toxic effect. Note that at the moment there are no data on the systematic infusion of solutions of halogenated taurine derivatives. However, it is known that these compounds, including NCT, are unstable and can be inactivated by transhalogenation or dehydrohalogenation, which is especially pronounced in the presence of an organic load, the role of which in our experiment is played by blood [15]. Thus, the absence of toxic effects may be due not only to the safe nature of NCT itself but also to its rapid metabolism. For a more complete analysis of the pharmacological effect of the described infusions, additional pharmacokinetic studies are required. Another limitation of our study is the lack of data on the effect of such infusions on the activity of the antioxidant system’s enzymes and other markers that would allow assessing the degree of oxidative stress provoked by the administration of a chlorine-active compound in experimental animals. 

Given the planned use of the studied NCT solution as a detoxifier, the results obtained by us of its administration against the background of simulated endotoxicosis are of particular interest. As can be seen from Table 10, the method chosen by us for the formation of pronounced endotoxicosis in animals was effective. The activity of transaminases increased significantly, which was similar to the data described in [72] and is an unambiguous marker of liver damage [73]. An increase in the concentration of MDA indicates significant oxidative stress, because it is a product of peroxidation of polyunsaturated fatty acids [74]; the same can be said for DC concentration. Superoxide dismutase is an enzyme of the first line of antioxidant protection, the most powerful scavenger of ROS, which converts superoxide anion into less toxic hydrogen peroxide and molecular oxygen. A decrease in the activity of SOD simultaneously with a sharp increase in MDA and DC are markers of an increase in the concentration of ROS in the body of rats injected with CCl_4_ and LPS, which leads to serious damage to various body systems. The data we obtained show that both chlorine-active compounds administered to rats—NCT and sodium hypochlorite—significantly change the level of all the listed markers towards normalization in relation to animals of the control group. This is probably caused by further oxidation of lipoperoxides and other ROS with them due to a higher oxidation potential. Note that taurine also had a similar effect, although less pronounced, which is explained by its powerful antioxidant properties. The effect of NCT infusions on the level of SOD activity is interesting. Despite the fact that SOD isresistant to the action of oxidants more than other antioxidant enzymes, in particular hypochlorous acid, high concentrations of active chlorine lead to its neutralization [75]. In our case, as can be seen, when both chlorine-active compounds were administered to rats, the activity of SOD significantly increased compared to the control group. Probably, such infusions, on the one hand, reduce SOD concentration due to the direct chemical neutralization of the enzyme, but on the other hand they act as inducers of its expression, as was assumed in [76]. The complex of obtained data allows us to state that the NCT solution when administered intravenously can act as an effective detoxifying agent, which, together with the above-mentioned absence of toxic effects, multifunctionality, and availability, makes it promising for use in the therapy of a wide range of endotoxicosis of various etiologies. The shortcoming of this part of our research is the lack of data on pathomorphological changes in the internal organs of rats, which would allow us to draw more complete conclusions about the pharmacological effect of NCT solutions.

Summarized data obtained during the research are shown in Table 11.

## 4. Materials and Methods

### 4.1. Characteristics of the Studied NCT Solution

**Scheme 1:**
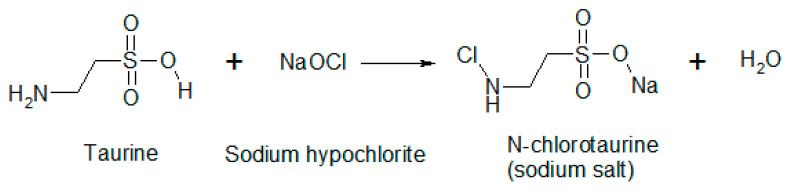
Chemical scheme of NCT synthesis.

In all studies, a solution of NCT called “Neoreodez” produced by LLC “Millipharm” and SE “Cherkasy-Pharma” (Cherkasy, Ukraine) was used, subsequently registered by the Ministry of Health of Ukraine for clinical research (Order of the Ministry of Health of Ukraine No. 334 from 11 June 2015, study codes CHP/NRD/SI/RN-II-01-03 from 10 June 2014). The basis of the preparation is a highly pure isotonic solution of sodium hypochlorite (NaOCl), obtained by special electrochemical technology [77] and later registered by the Ministry of Health of Ukraine as a disinfectant “SEKOBREN”. “Neoreodez” solution is prepared by dissolving taurine (QianjiangYongan Pharmaceutical Co., Ltd., Qianjiang, China) in “SEKOBREN”, diluted to a concentration of 660 mg/L of active chlorine, in accordance with the following chemical equation (Scheme 1):

The chemical analysis of the solutions was carried out by titrimetric methods developed earlier for sodium hypochlorite [78,79], as well as by spectrophotometric methods. The composition and properties of the NCT solution used are listed in Table 12.

As reference drugs in the experiments, the original solution of sodium hypochlorite “SEKOBREN” (660 mg/L active chlorine), 1320 mg/L taurine solution, and isotonic sodium chloride solution were used.

### 4.2. Characteristics of Experimental Animals

The research used white mature (age 12–16 weeks) Wistar Han female rats weighing 180–270 g obtained from the SPF vivarium of the Dnipro State Medical University.

The animals were kept in a special room with forced ventilation with a capacity of 5 volumes per hour without additional purification of the supply air. The room was constantly maintained at a temperature of 20–24 °C and a relative humidity of 45–60%. The lighting was artificial (12 h of light, 12 h of darkness). Animals were kept in 40 × 30 × 15 cm polycarbonate cages with removable metal grids on top, 5 individuals per cage. Sterilized, non-chlorinated food paper was used as litter, which was changed once every 5 days. Cells were washed and disinfected with the same periodicity. Animals had ad libitumaccess to granulated autoclaved Altromin feed (Lage, Germany) and water purified by reverse osmosis. Before each experiment, the animals were acclimatized for at least 10 days after being obtained.

All procedures to which the animals were subjected (administration of drugs, reproduction of the equivalent of endogenous intoxication, collection of material for research, etc.) fully complied with the principles of the European Convention for the Protection of Vertebrate Animals Used for Experimental and Other Scientific Purposes (Strasbourg, 1986), Directive No. 2010/63/EU on the protection of animals used for scientific purposes (2010), and local laws of Ukraine on the treatment of animals.

### 4.3. Method of Drugs Administration

Investigated NCT solution and all reference drugs were injected into the tail vein of rats. For this, the animal was fixed in a restrainer and its tail was immersed in warm (40 °C) water to dilate the vein. The injection site was dried and disinfected. Squeezing the vein near the root of the tail, a puncture was performed with a 28–29 G needle so that the needle passed superficially along the course of the vessel as far as possible. The studied drug was administered dropwise at a rate of about 1 mL/min in increasing doses. Doses were determined based on the circulating blood volume (CBV), which for rats of a given weight is approximately 12–15 mL [80] and in various experiments ranged from 0.1 to 2.0 CBV. The recommended maximum volume of fluids for a single intravenous slow bolus injection in adult rats is 10 mL/kg [81], so in cases when the volume of the drug was larger and could not be administered at once, the required dose was administered in parts over a period, which did not exceed 24 h [82].

**Figure 1:**
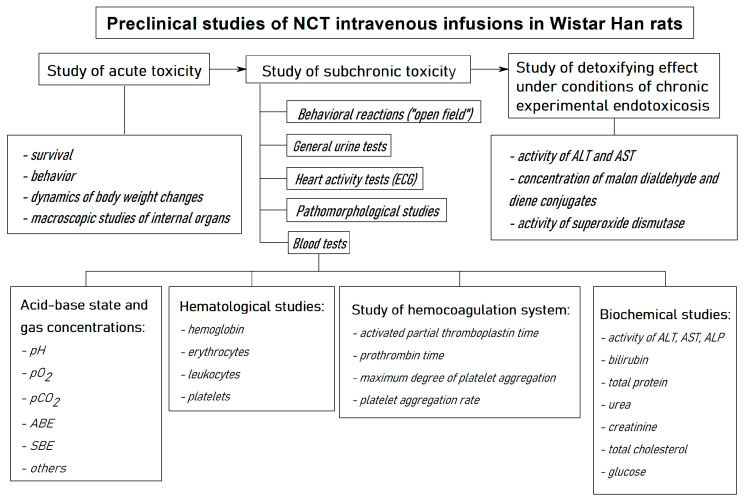
General research plan.

The general research plan is presented in Figure 1.

### 4.4. Study of Acute Toxicity of Intravenous Administration of N-Chlorotaurine Solution

The acute toxicity was studied on 48 white Wistar Han female rats weighing 180–220 g. After a preliminary 10-day quarantine, the animals were randomly divided into 8 groups of 6 animals each. Experimental animals (5 groups) were injected once with NCT solution in the amount of 0.1, 0.2, 0.5, 1.0, and 2.0 CBV, respectively. The animals of the reference groups (2 groups) were injected with 0.066% sodium hypochlorite and 0.132% taurine solution in a volume of 2.0 CBV. The control group was injected with 2.0 CBV of isotonic sodium chloride solution. The effect of the drugs was evaluated within 14 days after administration according to the following indicators: survival and behavior of animals (daily), dynamics of body weight changes (on the 7th and 14th day after administration), macroscopic studies, and weight coefficients of internal organs, namely the brain, liver, heart, spleen, lungs, kidneys, and adrenal glands (14 days).

### 4.5. Study of Subchronic Toxicity of Intravenous Administration of N-Chlorotaurine Solution

Subchronic toxicity of NCT when administered intravenously was studied in 48 rats, randomized into 4 groups of 12 animals each. Two groups of experimental animals were injected with NCT solution in the amount of 0.2 and 0.5 CBV, respectively, and animals of two control groups were injected with isotonic sodium chloride solution in the same corresponding volumes. The drugs were administered once every two days for 28 days. During the entire experiment, the animals were under daily supervision: appetite, weight of the animals, condition of the wool coat and mucous membranes, and behavior were evaluated. After the end of the course, the most comprehensive examination of animals was carried out using physiological, biochemical, and hematological tests and pathophysiological studies.

#### 4.5.1. “Open Field” Test

Behavioral reactions were studied using the “open field” test on the 28th day from the beginning of the drugs’ administration [83]. Motor activity was assessed by the number of crossed squares; passive defensive reactions—by the number of vertical racks; research activity—by the number of visits to the holes; emotional state—by the amount of washing (grooming) during 5 min of observation and defecation during 30 min of observation.

#### 4.5.2. Biochemical and Hematological Tests

Blood analysis of animals was carried out on the 28th day. Blood for the study of the concentration of basic blood cells and coagulation characteristics was taken from the tail vein of rats; for other biochemical studies, the animal anesthetized with thiopental was decapitated.

The number of erythrocytes, leukocytes, and blood platelets was counted in a hemacytometer. The preparation and staining of samples, as well as the determination of the blood formula, were carried out according to generally accepted methods [84]. Determination of blood hemoglobin was carried out by the hemichromic method using the biochemical analyzer HTI BioChem SA Chemistry Analyzer (High Technology Inc., North Attleborough, MA, USA) and a test kit manufactured by JSC “Reagent” (Kyiv, Ukraine). The activity of alanine aminotransferase (ALT), aspartate aminotransferase (AST), and alkaline phosphatase (ALP), as well as the content of total protein, creatinine, glucose, and urea in blood serum, were determined using the same analyzer and reagent kits for clinical biochemistry (High Technology Inc., North Attleborough, MA, USA). Determination of total cholesterol was carried out by Trinder’s enzymatic method [85] using a set of reagents produced by Filicit-Diagnostika (Dnipro, Ukraine). The evaluation of research indicators was carried out using the parameters and constants of the physiological norm [86,87].

#### 4.5.3. Study of Hemocoagulation System 

To study the effect of the NCT on the hemocoagulation system, the venous blood of animals was stabilized with a 3.8% solution of sodium citrate (ratio 9:1) and centrifuged at low speed (15 min at 100× *g*). As a result, plasma rich in platelets (platelet-rich plasma—PRP) was separated and collected in plastic tubes. To exclude contact activation of platelets, only plastic was used in the work. The rest of the blood was centrifuged again but at a higher speed (15 min at 2000× *g*). The upper layer formed after repeated centrifugation was platelet-poor plasma (PPP), which was also collected in plastic tubes. PRP was used to study the functional activity of platelets (the study of platelet aggregation). PPP was used to calibrate the scale of the optical density of the aggregometer and, if necessary, to dilute PRP to the standard cell content, which should be approximately 250 × 10^9^ cell/L. Also, PPP was used to perform basic coagulation tests.

The study of induced aggregation of platelets was carried out by the photometric method, recording changes in the optical density of PRP before and after the administration into it of a certain amount of aggregation inducer while observing the temperature regime and standard mixing [88,89]. The platelet aggregation analyzer “SOLARAP 2110” was used for this study. Adenosine diphosphate (ADP) solutions in final concentrations of 5 and 20 μM, as well as 2 mg/mL ofcollagen (MP Biomedicals, Santa Ana, CA, USA), were used as inducers. The maximum degree of aggregation and the rate of aggregation were determined. The measure of the aggregation process was a graphically recorded drop in the optical density of blood plasma as a result of the use of platelets in aggregates that are formed under the influence of aggregation inducers.

After preparing all the necessary solutions (PRP, PPP, aggregation inducers), they were kept in a thermostat at 37 °C for 3 min. Then, the optical density of PPP was determined on the aggregometer (its light transmittance in the relative scale of the device was taken as 100% aggregation). After that, a cuvette with PRP was inserted into the aggregometer, and the number of platelets in the plasma sample (if necessary, the platelet content was corrected by diluting PRP with platelet-free plasma) and its optical density (the light transmittance of PRP on the relative scale of the device was taken as 0% aggregation) were measured. After completing the calibration of the aggregometer, the study of the process of platelet aggregation under the influence of various inducers began. In total, 50 μL of the aggregation inducer solution was added to 450 μL of PRP in the cuvette, and the time of addition was recorded on the aggregometer. After initiation of the aggregation process, a drop in the optical density of the studied PRP sample was observed, which was registered by the device as an increase in the “degree of aggregation” indicator from 0% and above (maximum to 100%). As a result, 2 indicators were determined on the “degree of aggregation—time” curve: the level of the maximum rise of the curve (maximum degree of aggregation) and the steepness of the rise of the curve (speed of aggregation). The research time of each sample was 10 min.

Activated partial thromboplastin time (APTT) and prothrombin time (PT) were determined in citrated plasma using a SOLAR CGL2110 turbidimetrichemocoagulometer. A reagent was added to the studied plasma, which is an aqueous solution of ellagic acid (an activator of the internal coagulation pathway) in a complex with soy phospholipids [90]. In the process of measuring APTT, the time from the moment of addition reagent to the moment of clot formation was recorded. To study the PT, a water-soluble thromboplastin–calcium reagent was used, obtained from the brains of rabbits and certified according to the International Sensitivity Index [91]. The time from the moment of adding thromboplastin with calcium to the plasma to the moment of formation of a fibrin clot was measured.

#### 4.5.4. Study of the Acid–Base Balance of Rat’s Blood

In the mixed venous blood obtained from the right atrium of the rat, the partial tension of oxygen (pO_2_), saturation of blood with oxygen (sO_2_), partial tension of carbon dioxide (pCO_2_), and pH at a temperature of 37 °C were determined using a gas analyzer “Synthesis 15” (Instrumentation Laboratory, Bedford, MA, USA). The acid–base state of the blood was determined by Siggaard-Andersen nomograms according to the following parameters: real and standard excess of buffer bases (ABE/SBE), concentration of bicarbonate (HCO_3_^−^), total carbon dioxide (tCO_2_), and standard bicarbonate (SBC) [92].

#### 4.5.5. Urine Tests

Urine tests were performed on the 28th day from the beginning of the drug administration using standard diagnostic test strips DekaPHANLeuco (PLIVA-LachemaDiagnostika, Brno, Czech Republic). Biochemical analysis of urine for total protein content was performed on an HTI BioChem SA Chemistry Analyzer with appropriate reagents.

#### 4.5.6. Assessment the State of the Rat’s Heart Activity 

The functional state of the heart was assessed using the CardioLab+ computer complex with needle electrodes on the 28th day after the start of the NCT course. The analysis was carried out according to the time (duration of the PQ, QRS, QT, RR intervals) and amplitude (the size of the P, R, T waves) characteristics of the electrocardiogram in the II standard lead.

#### 4.5.7. Pathomorphological Studies

For pathomorphological studies, material was collected on the 28th day. The mass of organs (brain, liver, heart, spleen, lungs, kidneys, adrenal glands) was determined, and their histological studies were carried out. Organ fragments were fixed in 10% neutral formalin, dehydrated in alcohols of increasing concentration, and embedded in paraffin. Consecutive histological sections with a thickness of 8–10 μm, obtained using a Microm HM 325 microtome, were stained with hematoxylin and eosin. Optical viewing of stained preparations and photomicrographs was performed via Primo Star microscope (Zeiss, Oberkochen, Germany).

#### 4.5.8. Evaluation of Detoxifying Properties of NCT Solution under Conditions of Chronic Experimental Endotoxicosis

Modeling of experimental endotoxicosis was carried out by administering tetrachloromethane (CCl_4_) and bacterial lipopolysaccharide *E. coli* 0111B4 (LPS) to rats [93]. Twenty-eight rats randomized into four groups of seven rodents were used for this study. All animals were injected intragastrically with CCl_4_ (5 mL/kg body weight) 5 times a week, and 0.2 mL/kg LPS was additionally injected intraperitoneally on the 6th day. In the 1st (experimental) group of animals, every 5 days, the studied NCT solution was administered intravenously in a volume of 0.1 CBV. In the 2nd and 3rd groups, reference drugs (0.066% sodium hypochlorite solution and 0.132% taurine solution, respectively) were used in a similar manner. Animals of the fourth (control) group were injected with an isotonic sodium chloride solution. The experimental animals were removed from the experiment 30 days after the beginning of the simulation of the pathological process but not earlier than 12 h after the last manipulation. The assessment of the expressiveness of the therapeutic effect of the test sample and comparison drugs was performed by analyzing changes in the activity of cytolysis syndrome markers (ALT and AST), lipoperoxidation (malondialdehyde (MDA) and diene conjugates (DCs)), and antioxidant protection (superoxide dismutase SOD activity in the liver homogenate) caused by drug course application, for which Multiskan FC Microplate Reader (Thermo Fisher, Waltham, MA, USA) and appropriate ZELLX colorimetric kits (ZellBioGmBH, Lonsee, Germany) were used [94].

## 5. Conclusions

We have shown that both a single (in the amount of up to 2.0 CBV) and repeated (in the amount of up to 0.5 CBV for 28 days) intravenous administration of NCT solution with a concentration of 660 mg/L active chlorine to rats does not lead to the occurrence of obvious toxic effects. When such NCT infusions are used in animals with simulated endotoxicosis, a significant improvement in individual indicators of cytolysis and oxidative stress is observed. The complex of obtained data allows us to state that the NCT solution can act as an effective infusional detoxifying agent, which together with multifunctionality and availability makes it promising for use in the therapy of a wide range of endotoxicosis of various etiologies. 

## Figures and Tables

**Table 1:** Results of testing rats in the “open field” after 28-day administration of NCT.

Indicator	Animal Group		
	Control (0.5 CBV 0.9% NaCl)	NCT 0.2 CBV	NCT 0.5 CBV
Horizontal activity	21.6 ± 2.2	19.4 ± 1.5	18.2 ± 1.7
Vertical activity	6.1 ± 0.5	5.5 ± 0.4	5.1 ± 0.6
Research activity	4.6 ± 0.8	4.2 ± 0.5	3.9 ± 0.5
Grooming	3.2 ± 0.6	3.1 ± 0.5	2.9 ± 0.5
Defecation	1.4 ± 0.3	1.6 ± 0.3	1.9 ± 0.3

**Table 2:** Hematological indicators of peripheral blood of rats after repeated intravenous administrations of NCT solution for 4 weeks.

Indicator	Animal Group		
	Control (0.5 CBV 0.9% NaCl)	NCT 0.2 CBV	NCT 0.5 CBV
Hemoglobin, g/L	136.0 ± 3.55	137.3 ± 3.64	135.7 ± 3.66
Erythrocytes, ×10^12^ cells/L	4.41 ± 0.153	4.46 ± 0.159	4.39 ± 0.149
Leukocytes, ×10^9^ cells/L	8.84 ± 0.337	8.26 ± 1.028	8.52 ± 0.917
Platelets, ×10^9^ cells/L	242.4 ± 12.62	248.1 ± 13.22	250.0 ± 11.65

**Table 3:** Biochemical indicators of blood serum of rats after repeated intravenous administrations of NCT solution for 4 weeks.

Indicator	Animal Group		
	Control (0.5 CBV 0.9% NaCl)	NCT 0.2 CBV	NCT 0.5 CBV
ALT, МE/L	51.6 ± 2.84	52.4 ± 4.18	50.8 ± 2.23
AST, МE/L	126.4 ± 4.06	129.2 ± 4.26	131.4 ± 3.76
ALP, МE/L	284.6 ± 23.75	298.2 ± 26.88	308.6 ± 30.16
Bilirubin, mcmol/L	1.30 ± 0.13	1.43 ± 0.16	1.40 ± 0.15
Total protein, g/L	71.7 ± 1.6	73.7 ± 1.9	73.8 ± 1.7
Urea, mmol/L	7.67 ± 0.19	7.97 ± 0.16	8.00 ± 0.21
Creatinine, mg/L	4.34 ± 0.14	4.76 ± 0.38	4.52 ± 0.16
Total cholesterol, mmol/L	1.92 ± 0.13	1.87 ± 0.19	1.72 ± 0.16
Glucose, mmol/L	7.86 ± 0.14	6.44 ± 0.18	6.62 ± 0.21

**Table 4:** Activated partial thromboplastin time and prothrombin time of rat blood after 28 days of intravenous administration of *N*-chlorotaurine.

Animal Group	APTT, Second	PT, Second
NCT 0.2 CBV	21.1 ± 1.43	30.8 ± 1.33
NCT 0.5 CBV	20.8 ± 1.51	31.3 ± 1.53
Control (0.5 CBV 0.9% NaCl)	20.5 ± 1.66	29.8 ± 1.51

**Table 5:** The maximum degree of platelet aggregation (%) in rats after 28 days of intravenous NCT administration.

Animal Group	Inducer of Aggregation		
	ADP 5 μМ	ADP 20 μМ	Collagen 2 mg/mL
NCT 0.2 CBV	33.5 ± 3.9	35.4 ± 4.54	45.0 ± 4.24
NCT 0.5 CBV	27.8 ± 2.8	33.1 ± 3.46	44.6 ± 4.99
Control (0.5 CBV 0.9% NaCl)	44.2 ± 2.4	63.5 ± 1.12	68.9 ± 1.95

**Table 6:** Platelet aggregation rate (%/min) in rats after 28 days of intravenous NCT administration.

Animal Group	Inducer of Aggregation		
	ADP 5 μМ	ADP 20 μМ	Collagen 2 mg/mL
NCT 0.2 CBV	39.7 ± 4.59	29.0 ± 3.38	30.1 ± 3.14
NCT 0.5 CBV	28.8 ± 3.26	25.0 ± 4.54	21.9 ± 1.34
Control (0.5 CBV 0.9% NaCl)	59.2 ± 3.51	55.4 ± 2.96	58.1 ± 2.66

**Table 7:** Indicators of the acid–base state and gas composition of rat blood on the 28th day after repeated intravenous injections of NCT.

Indicator	Animal Group		
	NCT 0.2 CBV	NCT 0.5 CBV	Control (0.5 CBV 0.9% NaCl)
pН, units	7.35 ± 0.021	7.36 ± 0.019	7.33 ± 0.012
pO_2_, mm Hg	27.27 ± 0.69	28.03 ± 0.84	27.55 ± 0.75
pCO_2_, mm Hg	54.87 ± 0.77	54.70 ± 0.58	53.93 ± 0.69
SO_2_, %	27.48 ± 0.44	27.53 ± 0.50	27.71 ± 0.55
ABE, mmol/L	0.67 ± 0.30	0.25 ± 0.28	1.15 ± 0.39
SBE, mmol/L	1.01 ± 0.45	0.44 ± 0.32	1.74 ± 0.51
НCO_3_^−^, mmol/L	25.45 ± 0.44	24.78 ± 0.31	25.12 ± 0.38
tCO_2_, mmol/L	29.40 ± 0.36	28.98 ± 0.47	30.83 ± 0.41
SBC, mmol/L	26.02 ± 1.27	25.83 ± 1.70	26.87 ± 1.53

**Table 8:** Urine parameters of rats on 28th day after repeated intravenous NCT injections.

Indicator	Animal Group		
	Control (0.5 CBV 0.9% NaCl)	NCT 0.2 CBV	NCT 0.5 CBV
Volume, mL	4.72 ± 0.33	5.08 ± 0.29	4.80 ± 0.41
Color	Yellow	Yellow	Yellow
Transparency	Transparent	Transparent	Transparent
pН, unit	6	6	6
Density, g/mL	1.030 ± 0.012	1.033 ± 0.013	1.032 ± 0.009
Urobilinogen	None	None	None
Bilirubin	None	None	None
Microscopy of sediment			
Leukocytes	0–1	0–1	0
Cylinders	None	None	None
Epithelium	None	None	None

**Table 9:** The main indicators of the ECG of rats on the 28th day after repeated intravenous injections of NCT.

Indicator	Animal Group		
	Control (0.5 CBV 0.9% NaCl)	NCT 0.2 CBV	NCT 0.5 CBV
Heart rate, BPM	316.2 ± 10.26	414.2 ± 16.68	396.8 ± 14.36
Interval РQ, ms	42.6 ± 0.68	44.1 ± 1.54	43.6 ± 1.43
Interval QRS, ms	16.2 ± 0.95	15.8 ± 0.57	16.0 ± 0.68
Interval QRST, ms	60.3 ± 1.06	58.8 ± 1.44	59.6 ± 1.57
Interval RR, ms	178.3 ± 5.93	152.3 ± 7.26	156.4 ± 5.21
Amplitude Р, mV	0.057 ± 0.009	0.060 ± 0.012	0.061 ± 0.007
Amplitude R, mV	0.546 ± 0.049	0.564 ± 0.064	0.554 ± 0.060
Amplitude Т, mV	0.105 ± 0.022	0.098 ± 0.020	0.101 ± 0.018

**Table 10:** Indicators of the activity of cytolysis syndrome, lipoperoxidation, and antioxidant protection in simulated chronic endotoxicosis and its correction with intravenous NCT administration.

Indicator	Animal Group				
	Intact Animals	Control (CCl_4_ + LPS)	NCT 0.1 CBV	Taurine 0.1 CBV	NaOCl 0.1 CBV
ALT, МE/L	50.8 ± 3.36	158.7 ± 14.39	74.6 ± 6.46	112.3 ± 10.74	80.8 ± 7.43
AST, МE/L	90.9 ± 8.54	224.8 ± 21.45	109.7 ± 9.38	186.9 ± 17.44	113.2 ± 9.12
MDA, μmol/kg	3.16 ± 0.14	6.38 ± 0.16	4.18 ± 0.21	5.36 ± 0.18	4.58 ± 0.16
DC, units/g	0.97 ± 0.01	2.26 ± 0.04	1.44 ± 0.03	1.96 ± 0.04	1.58 ± 0.02
SOD, units/g	3.19 ± 0.164	1.78 ± 0.292	2.92 ± 0.154	2.14 ± 0.148	2.80 ± 0.146

**Table 11:** Summary table of data on the effect of intravenous infusions of NCT on Wistar Han rats.

Parameter	Results Obtained	Interpretation
Study of Acute Toxicity		
Survival	No deaths of animals observed	Absence of a pronounced toxic effect after the single administration of 0.1–2.0 CBV of the NCT test solution; LD50 > 2.0 CBV (>80 mg/kg)
General state	No differences found compared to the control group	
Behavior	Slight hypodynamia, salivation, slight increase in grooming at dosages above 1.0 CBV	
Body weight	No differences found compared to the control group	
Macroscopic changes in internal organs	No differences found compared to the control group	
Study of subchronic toxicity		
Survival, general state, body weight, macroscopic changes in internal organs	No differences found compared to the control group	High tolerability of repeated administrations of the NCT test solution in an amount of up to 0.5 CBV for 28 days
Open field test *	Slight dose-dependent decrease in the activity of animals; increase by 35% in frequency of defecation	Stimulation of defecation may be caused by influence of taurine as the metabolite of NCT [49]
Hematological studies	No differences found compared to the control group	No effect of repeated administrations of the NCT test solution on the blood cells ratio
Biochemical studies *	Slight dose-dependent increase in the levels of AST (up to 4%) and ALP (up to 9%); decrease up to 11% in the level of cholesterol; decrease up to 15% in the level of glucose	A slight tendency to activation of hepatocytolysis towards the end of the drug course may be assumed; the decrease in cholesterol is due to its oxidation by NCT [54,54]; the decrease in glucose is most likely due to the influence of free taurine [61,62]
Study of hemostasis system	No effect on the coagulation link of the rat hemostasis system observed; significant decrease in both the maximum degree and the speed of induced aggregation of platelets	Pronounced antiplatelet effect of NCT [69], which is also inherent in other chlorine-active drugs [63,64] and for free taurine [67,68]
Acid–base state and gas composition of blood *	Significant decrease in ABE (up to 4.4 times) and SBE (up to 3.95 times)	The effect is caused by a significant amount and amphoteric (with a predominance of acidic) properties of NCT [15]
Urine tests	No differences found compared to the control group	Presumed no effect on the urinary system
ECG *	Increase in heart rate (up to 31%)	Explained by previously described effects of free taurine [70,71]
Pathomorphological studies	No differences found compared to the control group	No damaging effects on the internal organs of experimental animals
Study of detoxifying activity under conditions of chronic experimental endotoxicosis		
Activity of transaminases	Significant decrease in activity of ALT (by 53%) and AST (by 51%)	Pronounced protective effect against hepatocyte cytolysis [73]
Markers of lipoperoxidation	Significant decrease in concentration of MDA (by 34%) and DC (by 36%)	Pronounced antioxidant properties of the test NCT solution [74]
Activity of antioxidant system	Significant (by 64%) increase in activity of SOD in the liver tissue	Putative stimulation of SOD expression leading to alleviation of oxidative stress [76]

* Only parameters that differ significantly from those for the control group of animals are given.

**Table 12:** Composition and properties of NCT solution used.

Parameter	Value
Appearance	Transparent colorless liquid with a weak characteristic odor
Active chlorine content, mg/L	660
*N*-chlorotaurine content, mg/L	1484
Free taurine content, mg/L	156
Sodium chloride content, g/L	8.32
Sodium chlorate content, mg/L	<10
Sodium chlorite content, mg/L	Not detected
Density at 20 °C, g/mL	1.01
pH at 20 °C, pН units	9.00–9.10

## Data Availability

The original contributions presented in this study are included in the article. Further inquiries can be directed to the corresponding author.
